# A Diagnostic Dilemma—Severe Hyperthermia and Rigidity in a Young Man with Polysubstance Use: A Case Report

**DOI:** 10.5811/cpcem.47071

**Published:** 2025-08-26

**Authors:** Joseph P. O’Brien, Matthew Carvey

**Affiliations:** * Denver Health, Department of Emergency Medicine, Denver, Colorado; † MetroHealth Medical Center, Department of Emergency Medicine, Cleveland, Ohio

**Keywords:** neuroleptic malignant syndrome, serotonin syndrome, sympathomimetic toxicity, hyperthermia, altered mental status, case report

## Abstract

**Introduction:**

Neuroleptic malignant syndrome (NMS) is a rare but life-threatening condition often associated with dopamine antagonist use. However, its overlap with other hyperthermic and toxidromic syndromes presents significant diagnostic challenges. We present the case of a 27-year-old man with severe hyperthermia, altered mental status, and diffuse rigidity, ultimately managed as possible NMS but with multiple differential diagnoses.

**Case Report:**

We describe a diagnostically challenging case of a 27-year-old male with an unknown medical history presenting with altered mental status, absence of personal identification, severe hyperthermia, and positive systemic inflammatory response syndrome criteria. The patient initially presented with hyperthermia (42.1 °C), tachycardia, tachypnea, diaphoresis, agitation, and rigidity. Initial lab findings demonstrated leukocytosis, elevated creatine kinase, metabolic acidosis, and rhabdomyolysis. Computed tomography ruled out acute anatomical abnormalities, while urine toxicology returned positive for amphetamines and cocaine. The patient required sedation, rapid sequence intubation, and dantrolene administration, which rapidly resolved his rigidity and hyperthermia and stabilized his vital signs. He was admitted to the intensive care unit, where supportive care, including antipyretics, hydration, and muscle relaxants led to gradual improvement. When the patient became less altered, he admitted to a history of aripiprazole use for schizophrenia, as well as daily amphetamine and cocaine use.

**Conclusion:**

This case underscores the importance of considering neuroleptic malignant syndrome in patients with atypical presentations, suspicion for comorbid psychiatric conditions, and substance use disorder. Timely diagnosis, discontinuation of the offending agent, and targeted therapies such as dantrolene are critical in preventing complications. We highlight the diagnostic challenges and management strategies for NMS in the context of a limited history and severe hyperthermia.

## INTRODUCTION

Hyperthermia presenting with concomitant altered mental status and neuromuscular findings represents a high-stakes diagnostic challenge in emergency medicine. Among the potential causes are neuroleptic malignant syndrome (NMS), serotonin syndrome, sympathomimetic toxicity, and malignant hyperthermia, which share overlapping clinical features yet differ markedly in their management. The stakes are high—failure to promptly identify and treat the correct syndrome can result in irreversible complications or death. Neuroleptic malignant syndrome is a rare and potentially life-threatening condition characterized by fever, muscular rigidity, altered mental status, and dysautonomia, primarily associated with exposure to dopamine-blocking agents.^1^ However, its presentation may be difficult to distinguish from other toxicologic and medical emergencies.

We report a case of an adult male initially presenting to the emergency department (ED) with severe hyperthermia, altered mental status, agitation, tremors, and diaphoresis with an unknown medical history. Given the diagnostic uncertainty and overlap with other hyperthermic syndromes, this case illustrates the critical importance of maintaining a broad differential and tailoring acute management based on syndrome recognition rather than premature diagnostic closure. We present this case not as a definitive diagnosis of NMS but as a clinical scenario highlighting the complexities of emergent toxicologic evaluation and the cognitive biases that can impact care. We discuss differential diagnoses, triage, and management strategies.

## CASE REPORT

A 27-year-old male presented to the ED from the local jail for concern of acute agitation and tremors. Review of systems was limited due to the patient’s altered mental status and agitated state. On initial evaluation, he exhibited muscular rigidity, tachycardia, tachypnea, and hyperthermia. Initial vital signs were as follows: temperature, 42.1 °Celsius; blood pressure, 117/77 millimeters of mercury; heart rate, 163 beats per minute; respiratory rate, 30 breaths per minute; and oxygen saturation, 97% on room air. The initial physical examination was notable for diaphoresis, rigid extremities with fine persistent tremors, severe agitation, and altered mental status. The patient also exhibited Kussmaul respirations and mild occiput bleeding. Of note, the patient’s pupils were equal, round, and reactive to light at 4 millimeters bilaterally.

Because the patient presented with an unclear medical history, further investigation was necessary. An electrocardiogram showed sinus tachycardia without evidence of ST-elevation myocardial infarction, QT interval prolongation or QRS complex widening. Initial laboratory results are presented in Table 1. A venous blood gas was consistent with mixed metabolic and respiratory acidosis.

Urine toxicology was noted to be positive for tetrahydrocannabinol, amphetamines, and cocaine. The patient’s acetaminophen and aspirin levels were unremarkable. Due to the blood found on his head upon initial examination, computed tomography (CT) of the brain without contrast was conducted and found negative for acute processes. A CT of the chest, abdomen and pelvis with contrast showed lower lobe opacities and dilated bowel loops concerning for possible aspiration pneumonia and ileus.

Due to the patient’s agitation and suspicion for possible sympathomimetic overdose initially, 2–4 milligrams (mg) of lorazepam was serially administered totaling 22 mg intramuscularly and intravascularly. The patient underwent rapid sequence intubation due to loss of airway protection with 150 mg propofol, 70 mg rocuronium, and post-intubation sedation with propofol and fentanyl. With a rectal temperature of 42.1 °C, the patient received 2.5 mg/kilogram dantrolene for concern of NMS or malignant hyperthermia. Within 1–2 minutes of administration, his vital signs improved, and the fine tremor and muscular rigidity resolved. The poison control center was consulted and recommended ongoing supportive care for suspicion of NMS.

*CPC-EM Capsule*

What do we already know about this clinical entity?

*Neuroleptic malignant syndrome, serotonin syndrome, sympathomimetic toxicity, and malignant hyperthermia share overlapping clinical features yet differ in their management*.

What makes this presentation of disease reportable?

*We offer insight into the cognitive challenges of managing critically ill, undifferentiated patients in the ED*.

What is the major learning point?

*This case underscores the complexity of assessing treatment response in critically ill patients where multiple interventions are administered concurrently*.

How might this improve emergency medicine practice?

*This case provides a framework for navigating diagnostic ambiguity when facing mixed toxidromes*.

The patient was accepted to the medical intensive care unit (MICU) and started on ceftriaxone and vancomycin for concern of a hip abscess found on physical exam, possible meningitis, or encephalitis. Blood cultures remained negative throughout his hospital stay, and antibiotics were discontinued. When the patient became less altered, his identification was obtained and revealed that he had presented to an outside hospital one day prior for a head injury, ultimately leaving against medical advice before a formal evaluation could be completed. The patient had also been seen two weeks earlier for right hip cellulitis and an abscess, for which he was prescribed doxycycline and keflex.

Additionally, he was evaluated by an outpatient psychiatrist two weeks prior for an underlying diagnosis of schizophrenia and a refill of his aripiprazole prescription, during which a history of amphetamine and cocaine use disorder was also noted. After consultation with psychiatry on MICU admission day 3 the patient’s aripiprazole remained held, and a diagnosis of NMS was suggested by both the MICU and psychiatry services. The patient’s symptoms improved over eight days, and he was discharged to jail with police.

## DISCUSSION

We describe a case of a 27-year-old man who presented with altered mental status, agitation, rigidity, hyperthermia, and unknown medical history. This case illustrates the diagnostic and therapeutic complexity of managing a critically ill patient in the absence of a clear history. The patient met many of the diagnostic criteria for NMS, including fever, severe muscle rigidity, diaphoresis, altered level of consciousness, tachycardia, elevated creatine kinase (CK), and leukocytosis.^2^ However, his rapid symptom onset and improvement following dantrolene administration occurred in the setting of multiple simultaneous interventions and is atypical for NMS, raising concern for premature diagnostic anchoring.

The differential diagnosis for altered mental status in the setting of dysautonomia is broad, and several alternative diagnoses were considered. Serotonin syndrome, sympathomimetic toxicity, malignant hyperthermia, heat stroke, and sepsis all remained in the differential. The classic features, onset, triggers, and notable labs for these differential diagnoses are presented in Table 2.^3^–^6^ Notably, the rapidity of symptom onset and the presence of polysubstance use (amphetamines and cocaine) are more consistent with sympathomimetic or serotonergic toxidromes, which are known to cause hyperthermia, agitation, tremor, and autonomic instability.^7^ Unlike NMS, these syndromes may present within hours of exposure, while NMS classically evolves more gradually over 1–3 days.

Distinguishing between these conditions can be clinically challenging. Serotonin syndrome, characterized by altered mental status, autonomic disturbances, and motor symptoms due to serotonin excess, shares many features with NMS. However, it can typically be distinguished by its clinical history, the absence of leukocytosis and elevated CK levels, and the presence of gastrointestinal symptoms, such as nausea, vomiting, and diarrhea, along with motor findings like tremor, ataxia, myoclonus, and hyperreflexia rather than rigidity.^5^ Sympathomimetic toxicity presents with mydriasis, agitation, tachycardia, and diaphoresis but rarely the profound rigidity observed here. Neuroleptic malignant syndrome, in contrast, features “lead-pipe” rigidity, hyporeflexia, and bradykinesia, often with recent or ongoing dopamine antagonist exposure. In this case, multiple features from different syndromes overlapped. The initial presence of fine tremors and diaphoresis, coupled with the patient’s toxicology screen, pointed toward a sympathomimetic component, while the muscular rigidity, elevated CK, and dopamine antagonist history raised concern for NMS. Malignant hyperthermia was considered less likely given the lack of triggering anesthetic exposure.

Infectious etiologies include meningitis, encephalitis, rabies, sepsis, or a brain abscess, although these typically present with a history of a prodromal viral illness, headaches, and meningeal signs and have characteristic findings on brain imaging and cerebrospinal fluid studies.^4^ Although we initiated antibiotic coverage and obtained blood cultures after the patient met systemic inflammatory response syndrome criteria, our suspicion for an infectious etiology was lower given the profound muscle rigidity. Heat stroke was considered in the differential diagnosis due to the presence of hyperthermia and altered mental status; however, several key features made it less likely.

The patient presented with profuse diaphoresis, which is more consistent with exertional heat illness but less typical of classic heat stroke where anhidrosis is common. Additionally, there was no reported environmental exposure to high temperatures or recent strenuous physical activity, which are hallmark triggers of heat stroke. The presence of severe muscle rigidity and elevated CK also pointed toward a neuroleptic or toxidromic etiology rather than thermoregulatory failure alone.^4^

Neuroleptic malignant syndrome has been primarily linked to first-generation antipsychotics such as haloperidol; however, second-generation agents including aripiprazole have also been implicated. although with lower incidence and severity.^1^ This case involved aripiprazole, a partial dopamine agonist with a lower affinity for D2 receptors compared to typical antipsychotics. The occurrence of NMS with aripiprazole supports existing literature indicating that any dopamine-modulating agent can precipitate this syndrome.^1^ Several case studies have reported NMS in patients with contributing factors such as dehydration, high-dose or rapidly escalating antipsychotic regimens, physical exhaustion, hyponatremia, iron deficiency, malnutrition, trauma, thyrotoxicosis, and comorbid substance use.^8^–^10^

This patient’s history of amphetamine and cocaine use may have exacerbated hyperthermia, tachycardia, and agitation, mimicking a sympathomimetic overdose. The distinction between these etiologies is critical, as management strategies differ. The cornerstone of NMS management is prompt discontinuation of the offending agent, supportive care, and targeted therapies such as dantrolene or bromocriptine. In this case, dantrolene administration rapidly resolved the patient’s rigidity and improved vital signs. However, it is important to note that use of dantrolene in NMS remains controversial, and multiple interventions were initiated simultaneously, including sedation with benzodiazepines, neuromuscular blockade with rocuronium, and supportive care. These agents alone can significantly reduce muscle activity, mitigate hyperthermia, and improve vital signs in patients with severe agitation and rigidity, regardless of the underlying toxidrome.

Given that the patient was intubated and pharmacologically paralyzed prior to the observed improvement, we cannot definitively attribute the change in clinical status to dantrolene alone. Rather, the temporal relationship should be interpreted with caution, as improvement may have resulted from a combination of therapies. This case underscores the complexity of assessing treatment response in critically ill patients where multiple interventions are administered concurrently.

This case also serves as a reminder of the risks associated with anchoring bias in emergency toxicology. Faced with severe rigidity, hyperthermia, and a known antipsychotic exposure, the clinical team initially anchored on a diagnosis of NMS. However, this early diagnostic focus may have limited consideration of alternative or overlapping etiologies such as serotonin syndrome or sympathomimetic toxicity, both of which were supported by elements of the presentation including rapid onset and stimulant exposure. Anchoring can lead clinicians to filter new information through the lens of a premature diagnosis, potentially overlooking critical features that do not fit the presumed syndrome. Acknowledging and challenging initial impressions is essential, especially in toxicologic cases with diagnostic ambiguity.

Neuroleptic malignant syndrome is a clinical diagnosis of exclusion, and in many cases a presumptive diagnosis must be made before all alternative explanations can be ruled out. This patient’s presentation and response to dantrolene suggest a possible overlap between syndromes, rather than a single causative pathology. The fact that his symptoms improved following dantrolene, in combination with propofol, lorazepam, intravenous fluids, and supportive measures, makes it difficult to attribute therapeutic success to any single intervention.

While this case does not present a novel toxidrome, its educational value lies in the structured exploration of diagnostic reasoning under uncertainty. The coexistence of both antipsychotic and stimulant exposures created a scenario where multiple life-threatening toxidromes—NMS, serotonin syndrome, and sympathomimetic toxicity—were plausible. Rather than highlighting a rare syndrome, this report offers insight into the cognitive challenges of managing critically ill, undifferentiated patients in the ED. It emphasizes the need to avoid cognitive pitfalls like anchoring bias and illustrates the difficulty of interpreting clinical responses to overlapping therapeutic interventions. For emergency physicians, this case provides a framework for navigating diagnostic ambiguity when facing mixed toxidromes, reinforcing the importance of maintaining diagnostic flexibility and focusing on syndromic management principles.

## CONCLUSION

This case exemplifies the diagnostic uncertainty that often surrounds presentations of hyperthermia, rigidity, and altered mental status in the emergency department. While neuroleptic malignant syndrome was strongly considered, overlapping features with serotonin syndrome, sympathomimetic toxicity, and sepsis complicated the clinical picture. The patient’s rapid clinical improvement after dantrolene administration occurred in the context of simultaneous neuromuscular blockade and aggressive supportive care, making causality unclear. Rather than affirming a single diagnosis, this case highlights the importance of maintaining a broad differential, recognizing the limitations of clinical tools, and approaching such presentations as diagnostic dilemmas. Avoiding premature anchoring can help ensure that emergent management strategies are appropriately inclusive and responsive to evolving clinical data.

## Figures and Tables

**Table 1:** Lab values upon initial presentation of man with hyperthermia and altered mental status.

Laboratory value	Result	Reference range
White blood cell	13.5 × 10^3^/μL	4.5–11 × 10^3^/μL
Hemoglobin	13.4 g/dL	12–16 g/dL
Platelets	499 × 10^3^/μL	150–450 × 10^3^/μL
Sodium	148 mEq/L	135–145 mEq/L
Potassium	5.7 mEq/L	3.4–5.0 mEq/L
Creatinine	1.53 mg/dL	0.5–1.1 mg/dL
Blood glucose	95 mg/dL	70–110 mg/dL
Phosphorus	6.7 mg/dL	2.5–4.5 mg/dL
Lactate	>10 mmol/L	0.5–1.6 mmol/L
Bilirubin	0.9 mg/dL	0.3–1.0 mg/dL
Alkaline phosphatase	62 U/L	33–136 U/L
Aspartate aminotransferase	64 U/L	9–39 U/L
Alanine aminotransferase	92 U/L	10–52 U/L
Lipase	164 U/L	0–160 U/L
Creatine kinase	295 U/L	22–198 U/L
pH	7.13	7.35–7.45
pCO_2_	34.2 mm Hg	35–45 mm Hg
Bicarbonate	22 mEq/L	22–26 mEq/L

*mmHg*, millimeters of mercury; *mg/dL*, milligrams per deciliter; *mEq/L*, milliequivalents per liter; *mmol/L*, millimoles per liter; *g/dL*, grams per deciliter; *pCO_2_*, partial pressure of carbon dioxide; *U/L*, units per liter; μ*L*, microliter.

**Table 2:** Differential diagnosis of hyperthermia with altered mental status and rigidity.

Syndrome	Key Features	Onset	Typical Triggers	Notable Labs	Relevance to Case
**NMS**	Rigidity, hyporeflexia, AMS, autonomic instability	Gradual (1–3 days)	Dopamine antagonists (eg, antipsychotics)	↑ CK, ↑ WBC, mild ↑ LFTs	Aripiprazole exposure, rigidity, ↑ CK supports diagnosis
**Serotonin syndrome**	Tremor, clonus, hyperreflexia, GI symptoms	Rapid (within hours)	SSRIs, MAOIs, serotonergic agents	May see ↑ CK, no leukocytosis	Tremor and agitation present but no clonus or GI symptoms
**Sympathomimetic toxicity**	Agitation, mydriasis, diaphoresis, tachycardia	Rapid (minutes to hours)	Cocaine, amphetamines	Mild ↑ CK, metabolic acidosis	Positive for amphetamines/cocaine, but rigidity atypical
**Malignant hyperthermia**	Rigidity, tachycardia, acidosis	Immediate during anesthesia	Inhaled anesthetics, succinylcholine	↑ CK, ↑ K^+^, acidosis	No anesthetic exposure
**Sepsis/Encephalitis**	Fever, hypotension, AMS, possible meningeal signs	Variable	Infection (bacterial, viral)	↑ WBC, lactate, abnormal CSF	Antibiotic prophylaxis for prior hip abscess, rigidity unusual
**Heat stroke**	Hyperthermia, AMS, dry skin (classic) or diaphoresis (exertional)	Acute	Environmental heat, exertion	↑ CK, may see DIC	No known environmental exposure

*AMS* indicates altered mental status*; CK*, creatinine kinase; *CSF*, cerebrospinal fluid; *DIC*, disseminated intravascular coagulation; *GI*, gastrointestinal; *LFT*, liver function test; *K*, potassium; *MAOI*, monoamine oxidase inhibitor; *NMS*, neuroleptic malignant syndrome; *SSRI*, selective serotonin reuptake inhibitor; *WBC*, white blood cells.
